# Anthelmintic activity of acetone extracts from South African plants used on egg hatching of *Haemonchus contortus*

**DOI:** 10.4102/ojvr.v83i1.1164

**Published:** 2016-07-28

**Authors:** Gerda Fouche, Bellonah M. Sakong, Olubukola T. Adenubi, Elizabeth Pauw, Tlabo Leboho, Kevin W. Wellington, Jacobus N. Elof

**Affiliations:** 1 CSIR Biosciences, Pretoria, South Africa; 2 Department of Paraclinical Sciences, University of Pretoria, South Africa

## Abstract

The nematode, *Haemonchus contortus*, is responsible for major economic losses in the livestock industry. The management of parasites such as *H. contortus* has been through the use of synthetic parasiticides. This has resulted in the presence of residues in meat and milk, which affects food safety. The development of resistance to available anthelmintics coupled with their high cost has further complicated matters. This has led to the investigation of alternative methods to manage nematodes, including the use of plants and plant extracts as a potential source of novel anthelmintics. Acetone extracts were prepared from 15 South African plant species and their anthelmintic activity determined using the egg hatch assay (EHA). The leaf extract of *Cleome gynandra* had the best inhibitory activity (68% ± 3%) at a concentration of 2.5 mg/mL, followed by the stem extract of *Maerua angolensis* (65% ± 5%). The extracts had a relatively low toxicity on Vero cells determined by the MTT (3-(4,5-dimethylthiazol-2-yl)-2,5-diphenyltetrazolium bromide) cellular assay.

## Introduction

Livestock production in tropical and developing countries is severely hampered by gastrointestinal parasites (Adejinmi & Harrison 1997; Hounzangbe-Adote *et al*. 2005). The gastrointestinal parasitic nematode, *Haemonchus contortus*, also known as the barber pole worm, resides in the gut of sheep and other livestock. It accounts for about 80% of the global parasite afflictions of diseased animals (Arosemena *et al*. 1999) and is notorious for its high pathogenicity (Angulo-Cubillán *et al*. 2010).

In small ruminants, gastrointestinal nematodes have traditionally been managed by the use of synthetic anthelmintic compounds (Mendoza de Gives *et al*. 1998). The systematic application of anthelmintic drugs, in an effort to manage infections produced by *H. contortus*, has led to the emergence of resistant strains (Akhtar *et al*. 2000; Prichard 1994). There have been reports of parasite resistance to anthelmintic drugs in many countries (Melo, Bevilaqua & Reis 2009; Schnyder *et al*. 2005), and multiple anthelmintic resistance has reached extreme levels (Torres-Acosta *et al*. 2012).

Certain drugs may also cause problems such as food residues and environmental pollution (Hammond, Fielding & Bishop 1997). This global problem has caused severe losses in productivity and is also the main restricting factor for the livestock sector (Melo *et al*. 2009; Waller 1994). Novel alternative methods are thus required.

Research has been conducted on plant species as alternative anthelmintics to manage gastrointestinal infections in small ruminants (Adamu, Naidoo & Eloff 2013; Batista *et al*. 1999; Slomp *et al*. 2009). The use of anthelmintic plant extracts may be sustainable and environmentally acceptable and could provide an alternative to synthetic anthelmintics. Furthermore, anthelmintic plant extracts have a mixture of active principles that could act in synergy, yielding the anthelmintic effect and limit the development of resistance. This differs from commercial drugs, which usually have only one molecule acting on the parasite when not administered as a combination formulation. Resistance is therefore likely to develop more slowly in the natural product.

The aim of this study was to determine the *in vitro* anthelmintic action of acetone extracts from 15 South African plant species used traditionally to control parasites such as *H. contortus* using the egg hatch assay (EHA). We used only one concentration as a first step in selecting plant species for in-depth follow-up research. The toxicity of the acetone extracts was also determined against Vero cells.

## Materials and methods

### Plant material collection

Fifteen plants [*Aloe rupestris* Baker, *Antizoma angustifolia* (Burch.) Miers ex Harv., *Calpurnia aurea* ssp. *aurea* (Aiton) Benth., *Senna italica* subsp. *arachoides* (Burch.) Lock, *Cissus quadrangularis* L., *Clematis brachiata* Thunb., *Cleome gynandra* L., *Ficus sycomorus* L., *Hypoxis rigidula* Baker var rigidula, *Maerua angolensis* DC, *Monsonia angustifolia* E. Mey. ex A. Rich., *Pelargonium luridium* (Andrews) Sweet, *Schkuhria pinnata* (Lam.) Kuntze ex Thell., *Sclerocarya birrea* (A. Rich.) Hochst and *Tabernaemontana elegans* Stapf.] were selected on the basis of available literature and ethno-veterinary usage over many years at Council for Scientific and Industrial Research (CSIR) (unpublished data). These plants were collected from different locations in South Africa during the summer season.

### Production of dried, ground plant material

Plant material was dried in an oven at 30 °C – 60 °C followed by grinding to fine particles using a hammer mill.

### Preparation of the acetone extracts

The acetone extract was prepared by adding 200 mL of acetone to 20 g of each ground plant material that was stirred for 1 h. The extract was decanted and filtered, and the residue was re-extracted with the same volume of acetone once again for 1 h; the third time, the same volume of acetone was used but the mixture was stirred overnight. The extracts were combined and the acetone evaporated on a rotary evaporator. The yield that was obtained for each of the plant species is shown in Table 1.

**TABLE 1:** The plant and plant part used for the solvent extraction, plant family, solvent, the mass and percentage yield of extract obtained.

Entry	Plant and plant part used in extraction	Plant family	Solvent	Mass of extract (g)	Yield of extract (%)
1	*Aloe rupestris* (leaves)	Asphodelaceae	Acetone	1.0127	5
2	*Antizoma angustifolia* (roots)	Menispermaceae	Acetone	1.0619	5
3	*Calpurnia aurea* (leaves, flowers)	Fabaceae	Acetone	0.9409	5
	*Calpurnia aurea* (stems)	Fabaceae	Acetone	1.0491	5
4	*Senna italica* subsp. *arachoides* (Burch.) Lock (roots, leaves, fruit)	Leguminosae	Acetone	1.0920	5
5	*Cissus quadrangularis* (stems)	Vitaceae	Acetone	1.0063	5
6	*Clematis brachiata* (whole plant)	Ranunculaceae	Acetone	1.0430	5
7	*Cleome gynandra* (leaves)	Capparidaceae	Acetone	0.9699	5
8	*Ficus sycomorus* (bark, stems)	Moraceae	Acetone	1.0339	5
9	*Hypoxis rigidula* (bulb)	Hypoxidaceae	Acetone	1.1049	6
10	*Maerua angolensis* (leaves)	Capparaceae	Acetone	1.1714	6
	*Maerua angolensis* (stem)	Capparaceae	Acetone	1.0524	5
11	*Monsonia angustifolia* (whole plant)	Geraniaceae	Acetone	1.0013	5
12	*Pelargonium luridum* (whole plant)	Geraniaceae	Acetone	1.0100	5
13	*Schkuhria pinnata* (whole plant)	Asteraceae	Acetone	1.1115	5
14	*Sclerocarya birrea* (bark, root)	Anacardiaceae	Acetone	0.9109	5
15	*Sclerocarya birrea* (fruit)	Anacardiaceae	Acetone	1.0142	5
16	*Tabernaemontana elegans* (leaves)	Apocynaceae	Acetone	1.0023	5

### Egg recovery and preparation

The method used was based on the World Association for the Advancement of Veterinary Parasitology (WAAVP) guidelines described by Adamu *et al*. (2013). Briefly, *H. contortus* eggs were collected from sheep that were housed indoors on a concrete floor. Approximately 10 g – 15 g of sheep faecal pellets were crushed in water to form a slurry and cleared of organic debris by serially filtering it through sieves of pore sizes 150 µm, 63 µm and 20 µm. The eggs were collected on a 20-µm sieve and washed off with a 40% sugar solution (density 1.18) into 50-mL centrifuge tubes. The tubes were then centrifuged for 5 min at 1000 rpm to separate the floating eggs from other debris. The supernatant was decanted on a 20-µm sieve, and the eggs were washed off with water and collected in a 500-mL container.

The concentration of eggs in the egg suspension was determined by counting the eggs using a microscope and a McMaster. The egg concentration was subsequently brought to a final concentration of 100 eggs per 200 µL. To avoid proliferation of fungi, 5 µg amphotericin B solution (Sigma, Germany) was added per millilitre of egg suspension.

### Egg hatch assay

The *in vitro* EHA was based on the procedure described by Adamu *et al*. (2013), which is based on the method described by Coles *et al*. (1992). The egg suspension (200 µL) containing ~100 fresh eggs were distributed into each well of a 24-flat-bottomed microtitre plate. The same volume (200 µL) of the dried plant extract (5 mg/mL) dissolved in solvent was added to each well. Albendazole was used as positive control, and the solvents were used as the negative controls. Albendazole was dissolved in 5% dimethyl sulphoxide (DMSO) in water and evaluated at various concentrations (0.008 μg/mL – 25 μg/mL). The plates were incubated for 48 h at 27 °C at 70% relative humidity. The experiment was replicated three times for each extract on the same plate. After incubation, the hatched larvae and unhatched eggs were counted using an inverted microscope under 20 × magnification. The percentage inhibition of egg hatching was calculated using the formula of Bizimenyera *et al*. (2006):

Egg hatch inhibition (%) = 100 (1 – number of larvae/number of larvae and eggs in water control)    [Eqn 1]

The results of the EHA are shown in Table 2.

**TABLE 2:** Mean inhibition percentages of the acetone extracts (2.5 mg/mL) on egg hatching of *Haemonchus contortus* from sheep and the toxicity values (LC_50_) against Vero cells.

Entry	Plant name and part used	Mean egg hatch inhibition (%) ± s.d.	Toxicity against Vero Cells LC_50_ (µg/mL) ± s.d.
1	*Cleome gynandra* (leaves)	68 ± 3	553.61 ± 18.83
2	*Maerua angolensis* (stem)	65 ± 5	180.64 ± 3.4
3	*Monsonia angustifolia* (whole plant)	56 ± 6	120.37 ± 4.06
4	*Senna italica* subsp. *arachoides* (Burch.) Lock (roots, leaves, fruit)	55 ± 13	46.31 ± 2.89
5	*Aloe rupestris* (leaves)	47 ± 7	63.46 ± 11.00
6	*Tabernaemontana elegans* (leaves)	47 ± 7	32.35 ± 0.88
7	*Schkuhria pinnata* (whole plant)	41 ± 14	39.93 ± 1.80
8	*Antizoma angustifolia* (roots)	37 ± 16	43.59 ± 6.28
9	*Calpurnia aurea* (stems)	32 ± 20	223.97 ± 5.3
10	*Sclerocarya birrea* (fruit)	28 ± 23	214.79 ± 14.0
11	*Calpurnia aurea* (leaves, flowers)	27 ± 16	166.63 ± 7.97
12	*Pelargonium luridum* (whole plant)	25 ± 10	30.58 ± 3.40
13	*Maerua angolensis* (leaves)	25 ± 6	73.76 ± 0.27
14	*Ficus sycomorus* (bark, stems)	25 ± 5	172.94 ± 8.91
15	*Ficus sycomorus* (stem)	21 ± 14	48.74 ± 1.32
16	*Hypoxis rigidula* (bulb)	17 ± 20	64.04 ± 2.53
17	*Clematis brachiata* (*whole* *plant*)	*11* ± *15*	117.00 ± 4.08
18	2.5% DMSO	7 ± 17	N.D.
		14 ± 13	N.D.
19	Water	16 ± 10	N.D.
		16 ± 9	N.D.
20	Doxorubicin	N.D.	2.97 ± 0.016

Note: Albendazole was the positive control and recorded 100% egg hatch inhibition at all concentrations (0.008 μg/mL – 25 μg/mL) used, whilst 2.5% DMSO recorded < 10% inhibition.

N.D., not determined.

### Determining the toxicity of the plant extracts

The toxicity of the plant extracts were determined by using the method employed by Adamu *et al*. (2013): Vero African Green monkey kidney cells were obtained from a confluent monolayer and then trypsinised and seeded (0.5 × 103 cells/well) in a 96-well microtitre plate. This was followed by incubation overnight at 37 °C in 200 μL of 5% minimal essential medium (MEM, Highveld Biological, South Africa) and supplemented with 0.1% gentamicin (VirbacR) and 5% foetal calf serum (Adcock-Ingram) (Adamu *et al*. 2013). The media were replaced with 200 μL of the extracts (1 mg/mL, 0.1 mg/mL, 0.01 mg/mL and 0.001 mg/mL) after 24 h and incubated for another 5 days. Viability of cells was determined using the tetrazolium-based colorimetric MTT assay (3-5-dimethyl thiazol-2-yl-2, 5-diphenyl tetrazolium bromide) as described by Mosmann (1983) (Adamu *et al*. 2013). Basically, the medium in each well was removed, replaced with fresh medium and 30 μL (5 mg/mL) MTT in phosphate-buffered saline (PBS) followed by incubation for 4 h. The medium was then removed before washing the cells with PBS and before the addition of DMSO (50 μL) to dissolve any formazan crystals (Adamu *et al*. 2013). A Versamax microplate reader at 570 nm (path length 1 cm) was used to measure the absorbance of the wells. Doxorubicin was used as a positive control and tested at different concentrations. The negative control was a well containing cells without an extract. The percentage of cell viability was calculated relative to the pure growth. The LC_50_ value was calculated by determining the concentration of each plant extract resulting in 50% reduction of absorbance compared to untreated cells. Tests on the concentration of each extract were carried out in triplicate, and each experiment was repeated three times. The LC_50_ results are expressed as the mean ± standard deviation (s.d.) of the three replicates. A plant extract having an LC_50_ value > 20 μg/mL has an acceptable level of toxicity, whilst a value < 20 μg/mL is regarded as toxic (Kuete & Efferth 2010).

## Data analysis

Excel for Windows 7 was used to record the results produced in this study. Kinetica 5.0 (Thermo) using a sigmoid inhibitory model was used to calculate the LC_50_ values. The results are presented as the mean LC_50_ and the standard deviation of the mean.

## Results

### Extraction of plant material

Acetone was selected as extractant because it has many advantages over other generally used extractants (Eloff 1998). The extraction yield was very similar for the different plant species and plant parts and varied from 5% to 6% (Table 1).

### Effect of different solvents on *Haemonchus contortus* egg hatching

The dried extracts did not dissolve in water, and therefore the use of acetone, DMSO and Tween 80 on the hatching of eggs. The final concentration of the solvents in the well was 50.0%, 25.0%, 12.5%, 6.3% and 3.1%. Even 3.1%, the lowest concentration of acetone, inhibited 94.0% egg hatching. The lowest concentration of Tween 80 inhibited 19.0% egg hatching. DMSO concentrations of 25.0% and higher led to 100.0% inhibition of egg hatching. The best results were obtained with 3.1% and 6.3% DMSO leading to 13.0% and 19.0% inhibition of egg hatching, respectively. The results for the negative controls of water and PBS were 13.0% and 11.0%, indicating that 3.1% DMSO did not have a marked effect on the egg hatching. The lower degree of hatching with the negative controls probably indicates damage to the eggs during processing. Consequently, the extracts were dissolved in 6.0% DMSO leading to a 3.0% final concentration after adding the same volume of egg suspension.

### Determining the inhibitory activity of the plant extracts on *Haemonchus contortus* egg hatching

The two negative controls also had a degree of activity on the egg hatching. The results are presented in Table 2 as the mean egg hatch inhibition (%) and the standard deviation of the mean. At 2.5 mg/mL, extracts of *C. gynandra* (leaves), *M. angolensis* (stem), *M. angustifolia* (whole plant) and *S. italica* subsp. *arachoides* (roots, leaves and fruit) had a mean inhibition rate of between 55% and 68% which was much higher than the water and DMSO negative controls. Our choice of 2.5 mg/mL appeared to be a good concentration because only four species had what would have been an LC_50_ in the order of 2.5 mg/mL. Albendazole, the positive control, recorded 100% inhibition at the lowest concentration, 0.008 µg/mL. The extract of *C. brachiata* (whole plant) had the lowest activity (11%), which was even lower than the water control group (16%). The activity of the plant extracts was so much lower than that of albendazole that the feasibility of using plant extracts can be questioned. Plant extracts may be more active in the larval development assay (LDA).

### Determining the cytotoxicity of the plant extracts

The tetrazolium-based (MTT) colorimetric assay (Mosmann 1983) was used to determine the viability of Vero African Green monkey kidney cells in the presence of each of the plant extracts and the results are shown in Table 2.

From these results, it is apparent that none of the plant acetone extracts were as toxic as doxorubicin (2.97 µg/mL ± 0.016 µg/mL = 5.12 µM ± 0.028 µM). The leaf extract of *C. gynandra* was the least toxic (LC_50_ = 553.61 µg/mL ± 18.83 µg/mL) followed by the stem extract of *C. aurea* (LC_50_ = 223.97 µg/mL ± 5.4 µg/mL), the fruit extract of *S. birrea* (LC_50_ = 214.79 µg/mL ± 14 µg/mL), the stem extract of *M. angolensis* (LC_50_ = 180.64 µg/mL ± 3.5 µg/mL) and the bark and stem extract of *F. sycomorus* (LC_50_ = 172.94 µg/mL ± 8.91 μg/mL). The whole plant extract of *P. luridum* (LC_50_ = 30.58 μg/mL ± 3.40 μg/mL) was the most toxic of all the plants against Vero cells.

## Discussion

The EHA is an *in vitro* assay used to evaluate the anthelmintic activities of natural products. The capacity to reduce egg hatching could help to modulate the risk of parasitism by limiting the infectivity of pastures grazed by ruminants (Max 2010).

The aim of this study was to determine the inhibitory activity of the acetone extracts of 15 plant species on egg hatching of *H. contortus* in order to select the most promising plant species that could control the nematodes in the animal gut for further study. In previous studies, it was found that aqueous extracts contained few compounds, had very low biological activity (Eloff, Famakin & Katerere 2005; Kotze & Eloff 2002) and had low or negligible anthelmintic activity (Bizimenyera *et al*. 2006; Worku, Franco & Miller 2009). Acetone was therefore selected as an appropriate extractant because it is miscible with organic and aqueous solvents, non-toxic to bacteria and fungi, and also has the capacity to extract a wide range of polar compounds (Eloff 1998). As shown in Table 2, it is evident that the extracts of four plant species had anthelmintic activity (inhibitory activity above 50%) at the concentration tested.

*Cleome gynandra* leaf extracts had the best anthelmintic activity with an egg hatch inhibition of 68% ± 3% and low toxicity (LC_50_ = 553.61 µg/mL ± 18.83 µg/mL) on Vero cells. Our results are in agreement with that of other researchers who have also reported on the anthelmintic activity of *C. gynandra*. Two authors (Jadhav, Ghawate & Bhamber 2011; Thenmozhi *et al*. 2014) used the unverified assumption that the Indian adult earthworm (*Pheretima posthuma*) could be used as a model for the activity of *C. gynandra* (syn. *Gynandropsis pentaphylla*) extracts against intestinal roundworm parasites of human beings because of its anatomical and physiological resemblance. They also used a physiologically non-relevant high concentration of 25 mg/mL and concluded that these extracts had potent anthelmintic activity when it killed earthworms after 53 min without examining any helminths. Sowunmi and Afolayan (2015) also did a phytochemical analysis of the acetone extract of different parts of *C. gynandra.* The polyphenolic contents of the various parts of the plant were significantly high. Leaf acetone extracts of *C. gynandra* had the highest concentration of total phenolics (126.79 mg/g ± 0.55 mg/g), flavonoids (40.58 mg/g ± 0.06 mg/g) and flavanols (42.41 mg/g ± 0.05 mg/g), whilst the stem extract had the highest amount of proanthocyanidins (419.01 mg/g ± 0.67 mg/g) compared to the leaves (403.29 mg/g ± 0.89 mg/g) and fruits (107.18 mg/g ± 0.59 mg/g). The low concentration of saponins and alkaloids suggests that this plant may have low toxicity (Sowunmi & Afolayan 2015). This suggestion is supported by the low toxicity we observed against Vero cells (LC_50_ = 553.61 µg/mL ± 18.83 µg/mL).

Alcohol and aqueous extracts from the leaves of *Cleome viscosa* Linn were also investigated for their anthelmintic activity against the adult Indian earthworm, *P. posthuma*, as well as *Ascaridia galli*. Three concentrations (50 mg/mL, 100 mg/mL and 150 mg/mL) of each extract were studied, which entailed the determination of time of paralysis and time of death of the worm. Both the extracts had significant anthelmintic activity at the highest concentration of 150 mg/mL. The water leaf extract had weaker activity than the methanolic leaf extract, and both extracts caused paralysis and death of worms. Phytochemical screening of the methanol extract showed that anthraquinone glycosides, phenolic compounds and steroids were present in *C. viscosa* Linn, whilst in the aqueous extract glycosides and phenolic compounds were present. Flavonoids were identified as being one of the chemical constituents amongst the phenolic compounds in the crude extracts. Polyphenolic compounds are known for their anthelmintic activity (Kaushik, Katiyar & Sen 1974; Lal *et al*. 1976; Szewezuk, Mongelli & Pomilio 2003). Synthetic phenolic anthelmintics such as niclosamide, oxyclozanide and bithionol interfere with energy generation in helminth parasites by uncoupling oxidative phosphorylation (Bate-Smith 1962; Martin 1997; Tandon *et al*. 1997). The phenolic content may therefore have produced similar activity in the extracts of *C. viscosa* Linn and *C. gynandra*.

The second best egg hatch inhibition of 65% ± 5% was by *M. angolensis* (stem) extract that had low toxicity (LC_50_ = 180.64 µg/mL ± 3.5 µg/mL) on Vero cells. Phytochemical screening of the methanolic extract of the stem bark found that glycosides, tannins, saponins, terpenes, flavonoids, carbohydrates, proteins and alkaloids were present in *M. angolensis* (Ayo *et al*. 2013; Meda *et al*. 2013; Pl@ntUse). These compounds could also be present in the acetone stem extract tested in this study. It has been reported that *Maerua edulis* (Gilg and Gilg-Ben.) DeWolf and *Maerua subcordata* (Gilg) DeWolf have been used in traditional anthelmintic remedies in Kenya to treat sheep infected with *H. contortus* (Gakuya 2001). In this study, aqueous extract from both unground and ground material of each plant material was prepared using boiling water. Twenty-one clinically healthy sheep of mixed breeds and sexes were randomly allocated to four treatment groups, four animals each. Faecal egg counts were performed for all the sheep. It was found that the crude extracts could control helminthoses to a reasonable extent and maintain the animal at a clinically healthy state. The Brine shrimp assay was used to detect bioactivity in the water, chloroform and methanol extracts of *M. subcordata* and *M. edulis.* The chloroform extract was the most toxic to the Brine shrimps compared to the water and methanol extracts (Gakuya 2001).

*Monsonia angustifolia* (whole plant) had an egg hatch inhibition of 56% ± 6% and also had low toxicity on Vero cells (LC_50_ = 120.37 µg/mL ± 4.06 µg/mL). Five compounds identified as aryl naphthalene lignans (5*-*methoxyjusticidin A, justicidin A, chinensinaphthol, retrochinensinaphthol methyl ether and suchilactone) were isolated during the fractionation of the organic (methanol-dichloromethane) extract of *M. angustifolia* (Khorombi 2006). Lignans are a group of naturally occurring phenolic compounds. The drug podophyllum, a lignan, is obtained from the dried root and rhizomes of two species of *Podophyllum* (Berberidaceae), the American species *Podophyllum peltatum* and the Indian species *Podophyllum hexandrum* (*Podophyllum emodi*). The European settlers reported using the root extensively, particularly as a cathartic and anthelmintic (Konuklugil 1995). Thus, the anthelmintic activity of *M. angustifolia* may be attributed to the lignans.

*Senna italica* subsp. arachoides (Burch.) Lock (roots, leaves, fruit) had an egg hatch inhibition of 55% ± 13% and had a higher toxicity on Vero cells (LC_50_ = 46.31 μg/mL ± 2.89 μg/mL) than that of *C. gynandra* (leaves), *M. angolensis* (stem) and *M. angustifolia* (whole plant) which also had anthelmintic activity.

*Aloe rupestris* (leaves) only had an egg inhibition of 47% ± 7% against *H. contortus* and low toxicity (LC_50_ = 63.46 ± 11.00) against Vero cells. It has been reported that other Aloe species such as *Aloe ferox* can affect *H. contortus* of goats negatively (Maphosa *et al*. 2010). The amino acids, saponins and sterols in *A. ferox* can disturb protein structure and therefore affect the growth and repair of the nematode body (Mabusela, Stephen & Botha 1990).

*Tabernaemontana elegans* (leaves) also only had an egg inhibition of 47% ± 7% and low toxicity (LC_50_ = 32.35 ± 0.88) against Vero cells. In Guadeloupe (French West Indies), another species, *Tabernaemontana citrifolia,* has traditionally been used as an anthelmintic preparation for ruminants (Marie-Magdeleine *et al*. 2010). Marie-Magdeleine *et al*. (2010) prepared aqueous, methanolic and dichloromethane extracts from the fruit, leaves and roots of *T. citrifolia* for testing on four developmental stages of *H. contortus*. The EHA, the LDA, the L3 migration inhibition assay (LMI) and the adult worm motility assay (AWM) were employed in the testing. From the tests it was apparent that there were significant effects for the different parts of *T. citrifolia* when compared to the negative control and that the differences depended on the parasitic stage. The efficacies on the larval development of *H. contortus* ranged from 88.9% to 99.8% for fruit, from 72.1% to 83.8% for roots and from 33.5% to 85.0% for leaves. For the methanolic extract a dose-dependent effect was observed. Alkaloid compounds are present in the different parts of *T. citrifolia* and may be responsible for the observed anthelmintic activity against *H. contortus* (Marie-Magdeleine *et al*. 2010). Another species, *Tabernaemontana coronaria,* was also investigated for anthelmintic activity against the Indian adult earthworm *P. posthuma.* It was found that the ethanolic extract of the leaves had potent anthelmintic activity (Pushpa *et al*. 2011).

Anthelmintic activity has also been documented for other plant species where an organic extract was also used in the determination of the anthelmintic activity (Ademola & Eloff 2010, 2^011^; Monteiro *et al*. 2010). Adamu *et al*. (2013) reported that *Heteromorpha trifoliate, Leucosidia sericea* and *Maesa lanceolata* had 100% inhibition, whilst *Clausenia anisata* had 80% inhibition at a concentration of 3.13 mg/mL in the EHA. Several other plant species have been documented as having anthelmintic activity. These are *Lantana camara* (Verbenaceae), *Tagetes minuta* (Asteraceae), *Mentha villosa* (Lamiaceae) (Albuquerque *et al*. 2007) and *Alpinia zerumbet* (Zingiberaceae) (Almeida 1993).

In this study, extracts of *C. gynandra* (leaves), *M. angolensis* (stem), *M. angustifolia* (whole plant) and *S. italica* subsp. *arachoides* (roots, leaves and fruit) only had inhibition activity between 55% and 68% at 2.5 mg/mL. Higher inhibitory activity for these extracts may have been obtained if they had also been evaluated at 3.13 mg/mL.

The kidney is one of the main sites of excretion in animals, and therefore, renal cells in culture were used as an indicator of toxicity for this study. These cells were explicitly chosen because of the favoured blood supply of the kidney and their high metabolic capacity. The results of the toxicity study were encouraging because the extracts had low toxicity (LC_50_ > 20 µg/mL) against Vero cells. The determination of cellular toxicity is valuable because it will give a good indication of whether *in vitro* toxicity is also an indicator of *in vivo* toxicity.

The results of this study are significant because egg hatch inhibition is an important method by which pasture contamination by animals can be reduced during grazing. Thus, there is the possibility that the plant material in this study can be administered as a feed to control helminths, although the concentrations required for efficacy were very high. Overall, the use of these botanicals could contribute to a helminth control programme.

## Conclusion

The acetone extracts of *C. gynandra, M. angolensis, M. angustifolia* and *S. italica* subsp. *arachoides* have some anthelmintic activity against *H. contortus* egg hatching. The activity of each of these extracts was lower than that of the positive control, albendazole. The most promising plant species is *C. gynandra,* which may be further studied to identify the active constituents responsible for anthelmintic activity.
