# Corrigendum: Anthelmintic activity of acetone extracts from South African plants used on egg hatching of *Haemonchus contortus*

**DOI:** 10.4102/ojvr.v88i1.1949

**Published:** 2021-07-27

**Authors:** Gerda Fouche, Bellonah M. Sakong, Olubukola T. Adenubi, Elizabeth Pauw, Tlabo Leboho, Mbokota C. Khosa, Kevin W. Wellington, Jacobus N. Eloff

**Affiliations:** 1 CSIR Biosciences, Pretoria, South Africa; 2 Department of Paraclinical Sciences, University of Pretoria, South Africa; 3 Agricultural Research Council – Tropical and Subtropical Crops, Nelspruit, South Africa

In the published version of this article, Fouche, G., Sakong, B.M., Adenubi, O.T., Pauw, E., Leboho, T., Wellington, K.W. et al., 2016, ‘Anthelmintic activity of acetone extracts from South African plants used on egg hatching of *Haemonchus contortus*’, *Onderstepoort Journal of Veterinary Research* 83(1), a1164. https://doi.org/10.4102/ojvr.v83i1.1164, the sixth author, Mbokota C. Khosa, was omitted from the ‘Authors’ and ‘Affiliations’ sections. The indicated author should be added as the sixth author, and the following affiliation should be added as his affiliation: Agricultural Research Council – Tropical and Subtropical Crops, Nelspruit, South Africa.

The Authors’ contributions section is hereby update to:

## Authors’ contributions

G.F. conceptualised the study. M.C.K. was involved in the collection of some of the plant material and in the preparation of the extracts used in the biological screening assays. G.F., K.W.W. and T.L. carried out the literature search and plant selection. T.L. prepared the plant extracts. J.N.E. and E.P. arranged for sheep to be infected, collected the eggs and guided the study. B.M.S. performed the egg hatch assay on the extracts. M.C.K. was also involved in the fractionation and isolation process in the natural product chemistry laboratory. O.T.A. screened the extracts for toxicity on Vero cells. K.W.W. wrote the first draft of the manuscript.

This correction does not alter the study’s findings of significance or the overall interpretation of the study results. The authors apologise for any inconvenience caused.

